# Feasibility and effectiveness of combination antiretroviral therapy in HIV-infected infants in Pietermaritzburg, South Africa

**DOI:** 10.1186/1758-2652-13-S4-P154

**Published:** 2010-11-08

**Authors:** D Van der Linden, S Purchase, NH McKerrow

**Affiliations:** 1 CHU Sainte-Justine, Paediatric Infectious Diseases Department, Montreal, Canada; 2 Edendale Hospital, Paediatric Department, Pietermaritzburg, South Africa; 3 Pietermaritzburg Metropolitan Hospitals Complex, Paediatric Department, Pietermaritzburg, South Africa

## Background

In the absence of treatment, 50% of HIV-infected children will die before 2 years of age. In a recent randomized controlled trial, a 76% decrease in mortality was observed in infants receiving early combination antiretroviral therapy [1]. The World Health Organization now recommends starting all HIV-infected infants on combination antiretroviral therapy on diagnosis [2]. However, few data are available outside a well-controlled research setting.

## Purpose of the study

To show the feasibility and effectiveness of treating HIV-infected infants in a state-funded clinic located in a poorly resourced South African township.

## Methods

A retrospective chart review was performed of all HIV-1 infected infants initiated on combination antiretroviral therapy (cART) between 1st May 2005 and 31st May 2008 at the Edendale Family Clinic, Pietermaritzburg, South Africa. All HIV-1 infected infants who were less than 1 year of age when antiretroviral therapy was initiated, and who had completed at least 6 months of treatment, were included. Weight for age Z scores, CD4 %, viral loads (VL) and haemoglobin were collected on initiation of treatment and at 6-monthly intervals thereafter. Virological success was defined as VL<25 copies/ml, immune recovery as CD4>25%. Z scores were analyzed using Epi-Info.

## Summary of results

Of 129 treated infants, 94 completed 6 months of cART; 60 completed 12 months and 39 completed 18 months of treatment. Mean age at initiation was 8 months (range 2.1-11.7). 77.2% had advanced disease (WHO Stage 3 or 4). The infants were severely malnourished, with a mean Z-score of -2.4 (range -6.1 - +0.8). Mean baseline VL was 4700 000 copies/ml. After 6 months of treatment, 52.3% of babies had an undetectable VL, with 75% having a VL of < 400 copies/ml. Viral suppression was achieved in 34 (56.9%) out of the 60 infants who completed 1 year of cART and 79.3% had a VL <400 copies/ml. Undetectable VL was found in 78.8% of the 39 children who received 18 months of treatment. Weight for age Z score increased from a mean of -2.4 (<3rd centile) at initiation of treatment to -0.3 (38th centile) for the children who received 18 months of cART. The CD4% increased from a mean of 16.5% at the start to 31.9% at 18 months.

## Conclusions

This study from a township in Kwazulu-Natal shows a good clinical, immunological and virological response to cART in HIV-infected infants, despite high baseline viral loads and advanced disease.
